# Discrete-time neural Markov models

**DOI:** 10.1186/s12874-026-02769-5

**Published:** 2026-01-22

**Authors:** Jesper Sundell, Ylva Wahlquist, Kristian Soltesz

**Affiliations:** Department of Automatic Control, Lund University, P.O. Box 118, Lund, 221 00 Sweden

**Keywords:** Markov models, Covariate modeling, Neural networks, Machine learning

## Abstract

**Background:**

Markov models can be used to describe the movement of individuals between states. In medicine, Markov models are used to describe, for example, disease progression and the effects of interventions on such progression. The models can further be used to identify medical risk groups and aid clinicians in clinical decision making. Although accurate individual predictions are crucial for utility on an individual level, modeling of relationships between covariates and transition probabilities includes assumptions regarding the distribution of the data and the functional form of the relationship limiting accurate prediction of individual risks. The purpose of this work was to develop a flexible framework for discrete-time Markov models enabling the use of neural networks to predict individual transition probabilities.

**Methods:**

The framework was implemented in Julia. Both real-world data and simulated data including two possible competing transitions, censoring and non-linear covariate relationships was used for model evaluation. Three models were evalauted: a model excluding covariates, a model including linear mappings between covariates and transition probabilities, and a model using neural networks for predicting individual transition probabilities based on covariates. Models were trained by minimizing the negative likelihood using mini-batching of data for efficient computations of gradients.

**Results:**

The neural network model outperformed the linear model in predicting individual transition probabilities. Both the negative likelihood and the mean squared error between observed and predicted individual transition probabilities were lower for the neural network model than for the linear model. Individual predicted transition probabilities were more evenly distributed around the line of identity for the neural network model than for the linear model indicating a better predicted functional form of the covariate mapping.

**Conclusion:**

A simple and flexible framework for development of discrete-time Markov models is described. Including neural networks to map the relationship between covariates and transition probabilities enables increased accuracy in predictions of individual risks and provides a tool to inform clinical interventions.

**Supplementary Information:**

The online version contains supplementary material available at 10.1186/s12874-026-02769-5.

## Introduction

Survival analysis or time-to-event analysis is concerned with the instantaneous risk of an event and the probability of survival at a given time. In medicine, events may, for example, include disease progression or other medical complications. The models may be used to evaluate the effect of drugs or patient-specific factors on the risk of interest to identify beneficial drug regimens or patient populations at risk.

Competing risks refer to when there is a susceptibility to multiple different events and where one event precludes other events. The competing risks framework is frequently used in medical research due to the natural occurrence of competitive risks in such a context [1]. For example, a patient may die from a disease or develop a comorbidity. Recently, there has been a growing interest in expanding traditional statistical methodology using machine learning algorithms [2].

In medical time-to-event data, patient measurements may commonly be discontinued prior to an event (i.e. dropout). The measurement time may also be limited so that no event has occurred in a proportion of patients when the measurement time ends thus resulting in truncation of the data. Such a limitation in the data is commonly referred to as right censoring. Another common limitation in medical time-to-event data is uncertainty in the actual time of an event. Rather than knowing the exact time of the event, a time interval, in which the event has occurred is known. Such an uncertainty is referred to as interval censoring. Both right censoring and interval censoring require consideration during model development.

When time-to-event data includes recurring events or multiple events of interest, the data can be modeled as transitions between a finite number of states [3]. In medicine, Markov models are an increasingly common choice for modelling of such data [4]. In addition to handling data with multiple outcomes, Markov models are also suitable for dealing with different types of censoring.

In Markov models, transition probabilities or transition rates are used to describe the movement of individuals between states. Markov models are based on the “memoryless” Markov assumption that the probability of transition from the present state to a different state only is dependent on the current state [3]. Transition probabilities may be constant (i.e. time homogeneous) or time non-homogeneous and can be linked to covariates.

Predictions from probabilistic models such as Markov models, may be used to aid clinicians in their decision making regarding patients. For example, a patient which is at a high risk of developing a comorbidity may require an adaptation in therapy or monitoring. For adequate utility of models, predictions of individual risks are hence crucial. In survival models and Markov models, risks are typically modeled as linear relationships to covariates [5–7]. Furthermore, risks can be allowed to vary over time by making distributional assumptions regarding the data. Making such assumptions can result in accurate predictions if the assumed distribution is true. However, misspecification may increase inaccuracy and cause biased predictions.

Artificial neural networks (ANNs) are powerful function approximators supported by the Universal Approximation Theorem [8]. Unlike linear models, ANNs offer flexibility and allow for modelling of complex covariate-parameter relationships. ANNs do not require user specified functions for covariate relationships or time variations in transition probabilities. However, in the absence of suitable regularization and validation, ANNs may overfit to the data used for parameter optimization, and thus loose the predictive capabilities. Linear models, on the other hand, may be overly simplistic if the covariate mappings are non-linear. Using linear models when the underlying relationship is non-linear can result in poor predictions of individual risks. If the model is used for clinical decision making, such poor predictions may ultimately harm patients. A framework allowing for modelling of complex covariate mappings will therefore provide a valuable tool for models used to guide decisions in health care.

Accurate predictions of disease related risks is an ongoing topic of research and a plethora of ANN-based methods have been proposed for modelling of survival data [9–15]. Methods incorporating machine learning algorithms to improve predictions by competing risks models have also recently gained attention [16–18]. However, to our knowledge, the incorporation of ANNs into discrete-time Markov models for individual predictions of transition probabilities has not been described.

In the present work, we developed a flexible discrete-time framework for Markov models which can handle commonly encountered limitations in medical data. The methodology considers both right censored and interval censored data and allows flexibility in mapping covariates to transition probabilities including the use of ANNs. Models are trained through stochastic mini-batching of data for efficient computations of gradients. The method is demonstrated by application on both real-world data and simulated datasets containing significant uncertainties including interval censoring.

## Method

The present framework is used for modeling of discrete time Markov chains. Models are thus under the “memoryless” Markov assumption and considers all transitions between states for the same individual independent.

We define $\lambda _{mn}$ as the probability to transition from state *m* to *n* during a time interval:

1 $$\begin{aligned} \lambda _{mn}=P(s_{k+1}=n |s_{{k}}=m), \end{aligned}$$

where $s_{k}$ is the state, in which, the individual resides at time $t = k$. All time intervals are assumed to be of equal length $\delta$. Throughout this work, we use $\delta =1$ so that $k = 0,1,2,...,k^*$. Here, the observation at $t = k^*$ is the time of transition to another state or the time when measurements discontinue (i.e. dropout).

All transition probabilities from one state are considered mutually exclusive. Transitions from one state are therefore jointly modeled. In other words, if we consider the transition probability matrix

2 $$\begin{aligned} P_{\lambda } = \left\{ \begin{array}{lll} \lambda _{11} & \lambda _{12} & \lambda _{13} \\ \lambda _{21} & \lambda _{22} & \lambda _{23} \\ \lambda _{31} & \lambda _{32} & \lambda _{33} \end{array}\right\} \end{aligned}$$

each row of the matrix can be modeled separately. The sum of the elements in each row is 1 since, during each time interval, an individual either remains in the current state or transitions to another state.

### Objective function

In this section, $\lambda _{mn}$ is denoted $\lambda$ for readability. Under the assumption that transitions occur according to an independent Bernoulli process, the probability of transition to one state at $t=k^*$ is

3 $$\begin{aligned} \mathcal {L}(\lambda |k^*)=\lambda ^\alpha \mathcal {S}(k^*-1) \end{aligned}$$

where

4 $$\begin{aligned} \alpha =\left\{ \begin{array}{ll} 1, & \text {if a transition occurred at}\ t=k^{*} \\ 0, & \text {no transition was observed and measurements are discontinued.} \end{array}\right. \end{aligned}$$

The survival function $\mathcal {S}(k^*-1)$ represents the probability of not transitioning up to $t=k^*$ and is formulated as

5 $$\begin{aligned} \mathcal {S}(k^*-1) = \prod ^{k^*-1}_{j=0}(1-\lambda ) = (1-\lambda )^{k^*}. \end{aligned}$$

If the exact time of transition is unknown and only an interval $I^* = (k_1^*,k_2^*]$, in which the occurred event is observed, i.e., $k^* \in (k_1^*,k_2^*]$, the likelihood can be constructed based on the survival functions for the lower and upper limits of the interval:

6 $$\begin{aligned} \mathcal {L}(\lambda |I^*)= & \mathcal {S}(k_{1}^*)-\mathcal {S}(k_2^*) \nonumber \\= & (1-\lambda )^{k_1^*}-(1-\lambda )^{k_2^*}, \end{aligned}$$

where $k_{1}^*$ and $k_{2}^*$ are the samples of the lower and upper limit of the interval $I^*$. The true transition time $k^*$ lies in the interval $I^*$.

Taking the logarithm of Eq. 3 gives the log-likelihood for one transition where the exact transition time is known, so that

7 $$\begin{aligned} l(\lambda |k^*) = \alpha \log \lambda + k^* \log \left( 1-\lambda \right) , \end{aligned}$$

and when only the transition interval $I^*$ is known, the log-likelihood becomes

8 $$\begin{aligned} l(\lambda |I^*) = \log \left( (1-\lambda )^{k_1^*}-(1-\lambda )^{k_2^*} \right) . \end{aligned}$$

If multiple events are of interest and the corresponding transition probabilities are modeled simultaneously, the survival function is modified to account for multiple parallel transition probabilities. The survival function for a transition from state *m* then becomes

9 $$\begin{aligned} \mathcal {S}(k^*-1)=\left( 1-\sum \limits _{n=2}^N\lambda _{mn}\right) ^{k^*}, \end{aligned}$$

where *N* is the number of states to which the individual can transition.

For interval-censored data, the likelihood function for $\lambda$ with an observed event becomes

10 $$\begin{aligned} \mathcal {L}(\lambda |I^*) = \sum \limits _{k=k^*_1}^{k^*_2} \lambda S(k-1), \end{aligned}$$

where the survival function from Eq. 9 is used. This gives the log-likelihood where the exact time of transition or time of discontinued measurements is known

11 $$\begin{aligned} l(\lambda |k^* ) = \alpha \log \lambda + k^* \log \left( 1-\sum \limits _{n=2}^N\lambda _{mn} \right) . \end{aligned}$$

For interval-censored data, the corresponding log-likelihood can be written as

12 $$\begin{aligned} l(\lambda |I^*) =\log \left( \sum \limits _{k=k^*_1}^{k^*_2} \lambda S(k-1) \right) . \end{aligned}$$

When multiple events and individuals are modeled simultaneously, the joint likelihood can be constructed by summing up the individual contributions from either Eqs. 11 or 12. We estimate transition probabilities $\lambda$ by maximizing the described likelihood.

### Models

To ensure that each $0\le \lambda _{mn,i} < 1$ and that the sum of all transition probabilities which results in leaving the present state is positive and less than one, we model the transition probability $\lambda _{mn,i}$ for an individual *i* from state *m* to *n* as

13 $$\begin{aligned} \lambda _{mn,i}=h\left( f_{mn}(\boldsymbol{\varphi }_i, \boldsymbol{\theta })\right) =\frac{\exp \left( f_{mn}(\boldsymbol{\varphi }_i, \boldsymbol{\theta }_{mn}) \right) }{1+ \sum _{n=2}^N\exp \left( f_{mn}(\boldsymbol{\varphi }_i, \boldsymbol{\theta }_{mn})\right) }, \end{aligned}$$

where $\boldsymbol{\varphi }_i$ is the covariate vector, $\boldsymbol{\theta }_{mn}$ is a parameter vector to be identified, and *N* is the number of competing states to which the individual can transfer from state *m*. Note that *h* in Eq. 13 simplifies to a regular sigmoid function when the number of possible states, to which an individual can transition, is one. Individuals who do not transition to another state, remain in state *m* with probability $\lambda _{mm,i}$.

In this work, three models were evaluated for each transition $\lambda _{mn}$:

- a constant model where $\lambda _{mn}$ was estimated as a constant without any dependence on $\boldsymbol{\varphi }$, i.e., $f_{mn}(\boldsymbol{\varphi _i},\theta ) = \theta _{mn}$,
- a linear model, $$f_{mn}(\boldsymbol{\varphi }_i, \boldsymbol{\theta }) = \boldsymbol{\varphi }_i \cdot \boldsymbol{\theta }_{mn}$$,
- an ANN, $f_{mn}(\boldsymbol{\varphi }_i, \boldsymbol{\theta }) = \mathcal {A}(\boldsymbol{\varphi }_i, \boldsymbol{\theta }_{mn})$ for mapping $\boldsymbol{\varphi }_i$ to $\lambda _{mn,i}$ where the parameters of the ANN are the parameter vector $\boldsymbol{\theta }_{mn,i}$.

The ANN consisted of two hidden layers with 50 nodes in each respective hidden layer. The swish function was used as activation function following each of the hidden layers.

### Data generation

**Fig. 1:**
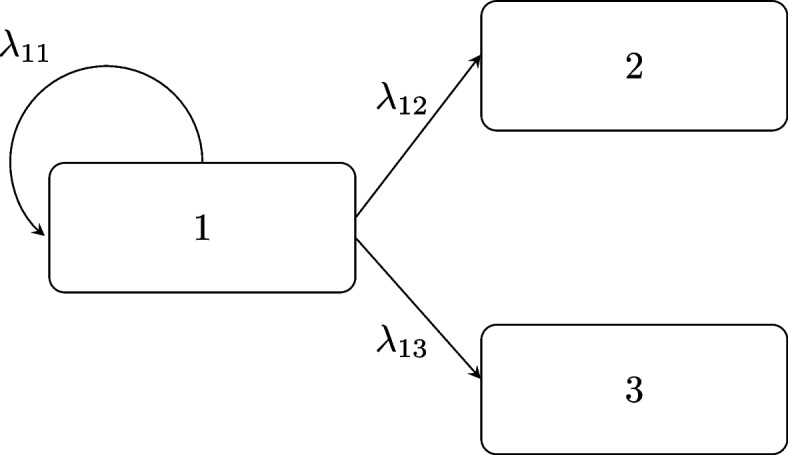
Example model consisting of three states. $\lambda _{mn}$ is the transition probability to transition from state *m* to state *n*

The present framework can be generalized to any number of states and transitions. However, to minimize computational times, a relatively small model was used for method demonstration. The model had three states, where the state *s* takes values in $\{1,2,3\}$. Transitions could only occur from one state to absorbing states. The model is illustrated in Fig. 1. All individuals started in state 1. From there they could transition to either state 2 or state 3 through transition probabilities $\lambda _{12,i}$ and $\lambda _{13,i}$ which are different between individuals, as indicated by subscript *i*. Individuals who do not transition to another state, stay in state 1 with probability $\lambda _{11,i}$.

In this example, uncertainty in the transition variable $t_k^*$ was considered. Uncertainty was added by sampling random noise from a Poisson distribution and generating an interval $I^*$, resulting in interval-censored data:

14 $$\begin{aligned} I^* = (k^*-\sigma _1,k^*+\sigma _2], \end{aligned}$$

where

15 $$\begin{aligned} \sigma _1 \sim \textrm{Poisson}(1), \quad \sigma _2 \sim \textrm{Poisson}(1) \quad \textrm{independently}, \end{aligned}$$

and $\sigma _1$ and $\sigma _2$ are nonnegative integers. Since the transition variable $t_k^*$ is geometrically distributed, the majority of transitions will occur within the first few discrete time steps. A Poisson rate parameter of 1 was therefore considered adequate for sampling of uncertainty.

A total of 100 training datasets with transition data for 3000 individuals based on the model was created. A corresponding 100 validation datasets including transition data for 1000 individuals were also generated. The validation datasets were used for model evaluation and were thus not included during training. Each dataset included discrete observations for each of these individuals of a transition from state $s_k$ at time point $t_k^*$. A covariate vector of two independent covariates $\boldsymbol{\varphi } = [\varphi _1, \varphi _2]$ was considered. When real data is considered, covariate values should be scaled between 0 and 1. However, for simplicity, covariate values for both covariates were generated using a uniform distribution truncated at 0.02 and 0.98, respectively.

To add further uncertainties to the studied example, a proportional error was added to the transition probabilities, so that

16 $$\begin{aligned} \lambda _{mn,i}= & g_{mn}(\boldsymbol{\varphi }_i) \cdot (1+ \epsilon _{\lambda _{mn}}), \nonumber \\ \epsilon _{\lambda _{mn}}\sim & \mathcal {N}(0, 0.05), \end{aligned}$$

where $\lambda _{mn,i}$ is the transition probability for an individual *i* from state *m* to *n* and $\epsilon _{\lambda _{mn}}$ is a normally distributed error, specific to this transition and the same over all individuals. The covariate vector of individual *i* is denoted $\boldsymbol{\varphi }_i$.

Underlying covariate functions for the transition probabilities were used to generate the transition times and were chosen based on the following conditions:

- the covariate mappings were non-linear
- the covariate functions included a non-monotonic mapping between a covariate and $\lambda$
- $$\lambda _{12,i}+\lambda _{13,i} \le 1$$.

The transition probabilities that were used to generate data were based on the following functions

17 $$\begin{aligned} g_{12}(\boldsymbol{\varphi }_i)=\ & \frac{1}{4}\left( \frac{0.5\varphi _{i,1}^3}{\varphi _{i,1}^3+0.027} +5\varphi _{i,2}(\varphi _{i,2}-0.9)(\varphi _{i,2}-0.8) + 0.05 \right) ,\nonumber \\ g_{13}(\boldsymbol{\varphi }_i)=\ & \frac{2}{3}\left( \exp {\left( \varphi _{i,1}^3 + 2\varphi _{i,1} -4\right) } +5\varphi _{i,2}(\varphi _{i,2}-0.9)(\varphi _{i,2}-0.8) + 0.15\right) , \nonumber \\ \lambda _{11,i}=\ & 1-(\lambda _{12,i}+\lambda _{13,i}), \end{aligned}$$

where the covariates $\boldsymbol{\varphi _i} = [\varphi _{i,1},\varphi _{i,2}]$ were scaled to be in the range of 0 to 1.

Generation of data consisting of individual transitions was performed by repeating trials of $\lambda _{11,i}, \lambda _{12,i}, \lambda _{13,i}$ by sampling from a categorical distribution with categories 1, 2 and 3 representing the model states. For each individual, sampling was repeated until category 2 or 3 was observed corresponding to a transition. The number of trials required until a transition was considered the time until a transition for that individual. A sample time of $\delta = 1$ was used. Each individual had a $50\%$ probability of being right censored. Right censoring was added to individual observations by subtracting a random number sampled from $\textrm{Poisson}(1)$ from the time until transition.

### Real-world data

To demonstrate the framework on real-world data, models were applied to data from the European Society for Blood and Marrow Transplantation (EBMT). The dataset is publicly available through the mstate package in R [19]. The dataset can be accessed by installing the package and loading the dataset referred to as ebmt2. Briefly, the dataset contains causes of death for 8966 transplanted patients and how long they remain alive following transplantation. Causes of death include relapse, graft-versus-host disease, bacterial infection, viral infection, fungal infection or other causes. Approximately $63\%$ of observations are right censored. All observed transitions end in an absorbing state and the transition probability matrix can thus be formulated as

18 $$\begin{aligned} P_{RD} = \left\{ \begin{array}{lllllll} \lambda _{11} & \lambda _{12} & \lambda _{13} & \lambda _{14}& \lambda _{15}& \lambda _{16}& \lambda _{17}\\ 0 & 1 & 0 & 0 & 0 & 0 & 0 \\ 0 & 0 & 1 & 0 & 0 & 0 & 0 \\ 0 & 0 & 0 & 1 & 0 & 0 & 0 \\ 0 & 0 & 0 & 0 & 1 & 0 & 0 \\ 0 & 0 & 0 & 0 & 0 & 1 & 0 \\ 0 & 1 & 0 & 0 & 0 & 0 & 1 \end{array}\right\} . \end{aligned}$$

The dataset contains four categorical covariates with two or three categories for each covariate. Covariates with two categories were set to 0 or 1 and covariates with three categories were set to 0, 0.5 or 1. Covariates are summarized in Table 1.

**Table 1:** Covariate distributions for the EBMT dataset presented as number of patients per category

Covariate	Number of patients
Disease subclassification (AML/ALL/CML)	3514/1870/3582
Donor-recipient gender match (No/Yes)	6758/2208
T-cell depletion (No/Yes/Unknown)	4390/1720/2856
Age at transplant (<20/20–40/>40)	1974/4800/2192

*AML* acute myeloid leukemia, *ALL* acute lymphoblastic leukemia, *CML* chronic myeloid leukemia

The dataset was divided into a training dataset ($80\%$ of observations) and a validation dataset ($20\%$ of observations). The validation data was excluded during training and was only used to evaluate model performance. Division of the data was performed so that all transitions were represented in similar proportions in both the training and the validation datasets.

### Training

Optimization of model parameters by maximization of the log-likelihood of Eqs. 11 or 12 using gradient descent-based algorithms is herein referred to as training. Training was performed using the adaptive learning rate algorithm ADAM [20]. Data batches consisting of data from 128 individuals were used for efficient calculations of gradients using back-propagation.

### Model evaluation

Model evaluation was performed using the validation datasets. Models were evaluated based on the value of the minimized negative log-likelihood and Brier score. Additionally, models applied to simulated data were compared by difference in mean squared error (MSE) between the true individual transition probability and the corresponding predicted transition probability. Models were compared by calculating the difference in each metric between the constant or linear model and the ANN model (e.g. $\Delta Loss = Loss_{linear}-Loss_{ANN}$). A positive difference is equivalent of a better predictive performance by the ANN model. For one dataset, observed and predicted transition probabilities were also plotted to demonstrate patterns in the predictions. Since predictions are biased by right censoring, the dataset used for the plot did not include right censored observations.

The Brier score was computed to evaluate models based on a metric which can be used without knowledge on the true transition probabilities [21]. The score approaches 0 as predictions improve. The Brier score was calculated by summing the squared distances between predicted and observed state occupations for all states *s* according to

19 $$\begin{aligned} BS_i(t)=\sum \limits _{s=1}^S (\pi _{i}(t)-\sigma _i(t))^2 \end{aligned}$$

where $BS_i$ is the Brier score of the $i^\textrm{th}$ individual evaluated at time *t* and $\sigma _i(t)$ is the observed state occupation probability. The individual predicted state occupation probability, $\pi _{i}(t)$, was derived according to the Chapman-Kolmogorov equation

20 $$\begin{aligned} \pi _i(t)=\pi _0P_i^t. \end{aligned}$$

Here, $\pi _0$ are the initial conditions and $P_i$ is the transition probability matrix for the $i^\text {th}$ individual. Since all individuals started in state 1, $\pi _0$ is the vector (1, 0, 0) for the simulated data and (1, 0, 0, 0, 0, 0, 0) for the EBMT data. The transition probability matrix contains all possible transition probabilities between the different states. If a certain transition is not possible, the corresponding element of the transition probability matrix is 0. Furthermore, if a state is absorbing, the probability to remain in that state is 1, and all probabilities of transitioning from that state are 0. Applied to the simulated data, *P* is thus

21 $$\begin{aligned} P = \left\{ \begin{array}{lll} \lambda _{11} & \lambda _{12} & \lambda _{13}\\ \lambda _{21} & \lambda _{22} & \lambda _{23}\\ \lambda _{31} & \lambda _{32} & \lambda _{33} \end{array}\right\} =\left\{ \begin{array}{lll} \lambda _{11} & \lambda _{12} & \lambda _{13}\\ 0 & 1 & 0\\ 0 & 0 & 1 \end{array}\right\} . \end{aligned}$$

To calculate the Brier score for interval censored observations, the mean of the Brier score for all possible transition times was derived:

22 $$\begin{aligned} BS_{i,I}=\frac{1}{t_2-t_1} \sum \limits _{t=t_1}^{t_2}BS_i(t). \end{aligned}$$

The total Brier score was simply the mean of all individual contributions to the total score. For the EBMT data, the Brier score was evaluated at five years following transplantation. For the simulated data, the Brier score was evaluated at $t=5$.

### Software

The present work was implemented in the Julia language (version 1.11). The Lux package (version 1.7) was used for model training. Code of the implementation can be found in the GitHub repository [22].

## Results

The total computational times for the ANN model were approximately 2-fold higher than for the constant model and the linear model for an equal number of training iterations. Computational times for all models are summarized in Table 2. The influence of total number of observations on predicted transition probabilities is illustrated in the supplemental material. The difference in the final loss values of the converged models and the mean squared error between the true individual transition probability and the corresponding predicted transition probability are summarized in Table 3.

**Table 2:** Computational times for the evaluated models presented as median ($95\%$ confidence interval)

Model	Time (seconds)
Constant model	54.0 (53.9–54.0.9.0)
Linear model	54.2 (54.2–54.3.2.3)
ANN model	93.4 (93.0–93.9.0.9)

*ANN* artificial neural network. All time values are based on 500 training iterations

**Fig. 2:**
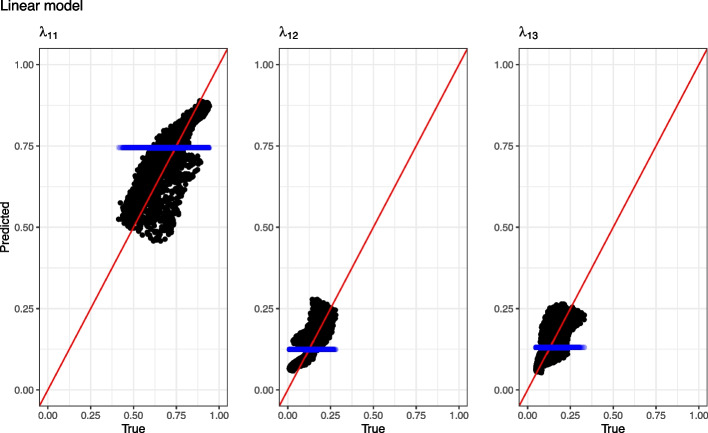
Observed versus predicted transition probabilities for the linear model for one dataset without right censoring. Red line is the line of identity. Blue points are transition probabilities predicted by the constant model

**Fig. 3:**
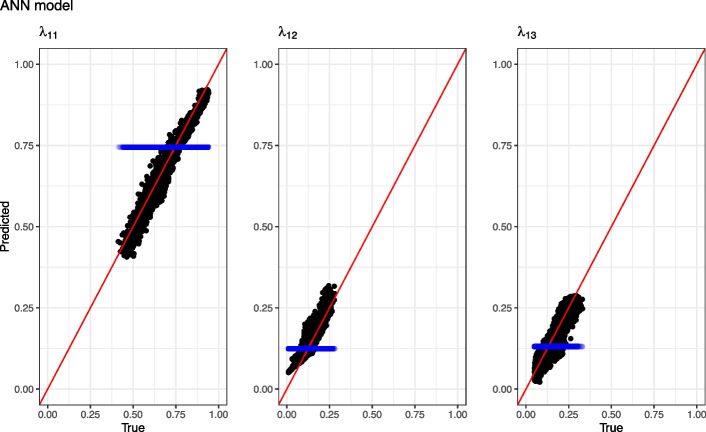
Observed versus predicted transition probabilities for the neural network model for one dataset without right censoring. Red line is the line of identity. Blue points are transition probabilities predicted by the constant model

Both loss value and mean squared error for all predicted transition probabilities were lower for the ANN than for the linear and constant models. The increase in prediction accuracy when using an ANN compared to the constant and linear models was most notable for $\lambda _{11}$. The Brier score was lower for the ANN than for the linear model and the constant model. The highest Brier score was achieved with the constant model. Individual true transition probabilities vs predictions for the linear model and the ANN model is illustrated in Figs. 2 and 3 respectively. For the ANN, the data points were closer and more evenly distributed around the line of identity compared to the linear model.

**Table 3:** Comparison between models based on 100 simulated datasets. Metrics are presented as median $(95\%$ confidence interval) difference from the ANN model

Metric	Constant vs ANN	Linear vs ANN
$\Delta$Loss value	61.3 (60.7–64.6.7.6)	7.9 (7–9.4.4)
$\Delta$MSE $\lambda _{11}$	0.029 (0.028–0.03.028.03)	0.0025 (0.0022–0.0031.0022.0031)
$\Delta$MSE $\lambda _{12}$	0.015 (0.015–0.017.015.017)	0.0016 (0.0015–0.002.0015.002)
$\Delta$MSE $\lambda _{13}$	0.013 (0.013–0.014.013.014)	0.0013 (0.0012–0.0018.0012.0018)
$\Delta$BS	0.085 (0.082–0.087.082.087)	0.023 (0.022–0.024.022.024)

*ANN* artificial neural network, *MSE* mean squared error, *BS* Brier score. $\lambda _{mn}$, probability of transition from state *m* to state *n*. Loss value is the minimized joint log-likelihood. Positive numbers indicate better performance by the ANN model

Both the linear model and the ANN model achieved predictive performance higher than that of the constant model in the EBMT validation data. The ANN model further outperformed the linear model. Performance metrics for the models applied to the EBMT data is summarized in Table 4. An example of difference in predicted state occupation probability for one individual using the three different models is illustrated in the supplementary material.

**Table 4:** Comparison between models applied to the EBMT validation data

Metric	Constant vs ANN	Linear vs ANN
$\Delta$Loss value	44.7	13.3
$\Delta$BS	0.011	0.0039

*ANN* artificial neural network, *BS* Brier score. Loss value is the minimized joint log-likelihood. Positive numbers indicate better performance by the ANN model

## Discussion

In this work, we describe a framework for discrete-time Markov models. We further describe how ANNs can be incorporated to model individual transition probabilities. The method considers censoring types commonly encountered in medical data. Although left-censoring was not included in this work, the framework can easily be extended to handle such observations. To our knowledge, this is the first described framework for discrete-time Markov models incorporating ANNs to model covariate relations.

A benefit of using ANNs is that no functional forms of covariate mappings or distributions of the data are assumed. Since the expectation is modeled, the highest accuracy gain using flexible models will occur for transition probabilities which are further away from the mode. In practice, this will result in more accurate risk predictions for individuals with more uncommon demographics. ANN-based models could, in extension, also improve identification of clinically relevant covariates since assumed functional forms can reduce the possibility of detecting relevant covariates.

In the present study, we demonstrated how ANNs can outperform commonly used linear models with regards to predicting individual transition probabilities. In the simulation study, the largest gain in predictive accuracy from using an ANN was for $\lambda _{11}$. Such an outcome is not surprising since all observations provide information on the probability of remaining in the current state and the survival function contributes to the likelihood irrespectively of which, if any, transition that occurs. Although being a derived parameter, the probability of remaining in the current state is thus also expected to be more accurately predicted when model flexibility increases. However, the benefit of using ANNs over simpler models will differ depending on multiple factors such as the number of observations, the dimensionality of predictors and the underlying relationship between covariates and transition probabilities. Model choice and modeling procedure will therefore require consideration depending on the data. Nevertheless, the accuracy in predicting $\lambda _{11}$ emphasize the benefit of jointly modeling multiple outcomes.

When applied to the EBMT data, the ANN model achieved a higher predictive performance than the linear model. The effect of a more accurate estimation of individual transition probabilities was exemplified by illustrating predicted state occupation over time for the different models. Overall, the ANN model predictions were further away from the predictions by the constant model than the predictions by the linear model indicating that the individual transition probabilities significantly deviates from the expectations. Such outliers are expected to be more accurately captured by an ANN than by a linear model and showcase an example of where more flexible models are beneficial.

Multi-state models are continuous-time generalizations of survival models for data with multiple outcomes. Multi-state models based on neural ordinary differential equations have been proposed to improve predictions by relaxing covariate-parameter relationship restrictions [23]. Such models are beneficial since the transitions between states are not assumed to be independent. However, implementation of multi-state models is complex and the resulting model can be difficult to communicate. Discrete-time Markov models, on the other hand, are simple to develop and a transition probability such as a monthly probability of disease progression is more intuitive to interpret than a transition rate. Clinical data is further discrete in nature due to visit-based collection of samples from patients inherently causing interval censoring. Moreover, discrete-time modelling provides a computationally efficient approach for estimation of transition probabilities. Notably, if the Markov assumption is not fulfilled, relaxation could be achieved by, for example, using the previous state as a covariate for the probability of transitioning to the next state. In the end, the choice of model type is a balance between adequacy and complexity.

In this work, we minimize the negative log-likelihood and the predictions are probabilities. Unlike regression problems, over-fitting may thus result in predictions far from the true underlying probabilities. To avoid over-fitting, we therefore used small networks with only two hidden layers. Using deeper or larger networks did not result in any significant reduction in loss function value (a comparison can be found in the supplementary material). Of note, the limited benefit of increasing network size and depth is specific to the simulated data and increasing the dimensions of the networks may result in additional predictive gain when applied to other datasets. Interestingly, using $\mathcal {L}_2$ regularization reduced model predictions to be close to equivalent to the predictions of the linear models. Although no benefit from regularization was found for this dataset, $\mathcal {L}_2$ and $\mathcal {L}_1$ regularization may improve generalizability for models and should be considered during model development [24].

A natural limitation of neural networks is the lack of model interpretability. Yet, equivalently to any other function mapping covariates to model parameters, the effect of covariates on risks can be simulated using an ANN. An ANN-based model may therefore inform interventions similarly to a linear model. Of note, simulations using an ANN outside of the studied covariate range should be performed with caution. Concerning generalizability of ANNs, symbolic neural networks have recently been proposed for the use of neural networks to create symbolic expressions for the functions relating covariates to model parameters [25]. Such an approach can be used in combination with the framework presented herein. Combining the two strategies may improve generalizability of the models and would indeed be interesting to explore further.

Many non-linear monotonic functions are relatively well approximated by linear functions. We therefore included one non-monotonic function in the covariate mappings to illustrate a case where ANNs excel in predictive performance compared to linear models. For simplicity, the same function was included for both transitions. In a clinical scenario, it is further likely that one covariate affecting the transition probability from a certain state will also affect other transition probabilities from the same state similarly. For example, if physical workout decrease the probability of developing a comorbidity, it will likely also have a positive effect on the probability of developing other comorbidities.

The increase in predictive performance gained by using ANNs over linear models is dependent on multiple factors such as the number of observations, underlying covariate functions, covariate interactions, and dimensionality. The effect of relevant factors on the gain in predictive performance from using neural Markov models would therefore be interesting to investigate further. Such an investigation would aid in understanding the boundaries of the conditions under which ANN-based models provide an advantage over traditional linear models.

## Conclusion

A discrete-time framework for Markov models including neural Markov models is presented. The framework can handle common limitations encountered in medical data such as censoring. The use of ANNs for individual predictions of transition probabilities may increase accuracy in predictions and thus provide more precise guidance for medical workers regarding patient interventions.

## Supplementary Information

Supplementary Material 1.

## Data Availability

Data is provided in the GitHub repository.

## Abbreviations

ANN: Artifical neural network
EBMT: European Society for Blood and Marrow Transplantation
MSE: Mean squared error
