# Supplementary material for: Discrete-time neural Markov models
Source: BMC Med Res Methodol. 2026 Jan 22;26:21. doi: 10.1186/s12874-026-02769-5 (PMC12857127; doi:10.1186/s12874-026-02769-5)
Supplement: Supplementary file 1 — Supplementary Material 1. [file 12874_2026_2769_MOESM1_ESM.pdf]

Supplement to Discrete-time neural Markov models

1 Predicted state occupation for one example patient

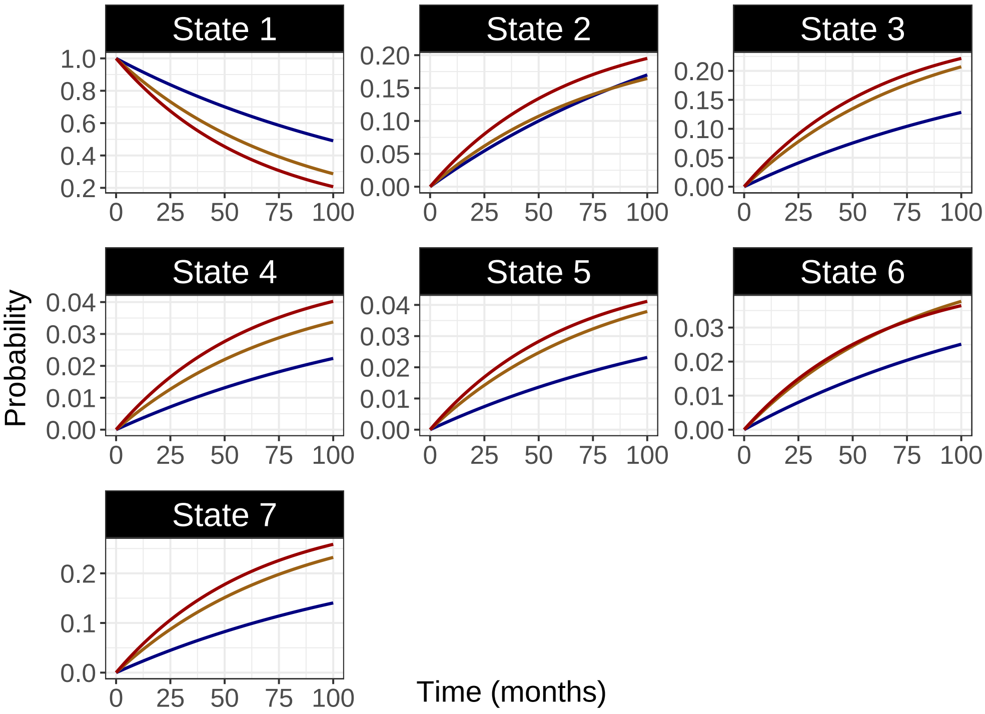

Fig. 1 Predicted state occupation probabilities for one patient in the EBMT dataset. Red line is the prediction by the ANN model, brown line is the prediction by the linear model and blue line is the prediction by the constant model.

2 Influence of number of individuals predictions

Note that datasets used to asses the influence of different number of individuals on predicted transition probabilities did not include right censored observations to avoid estimation bias.

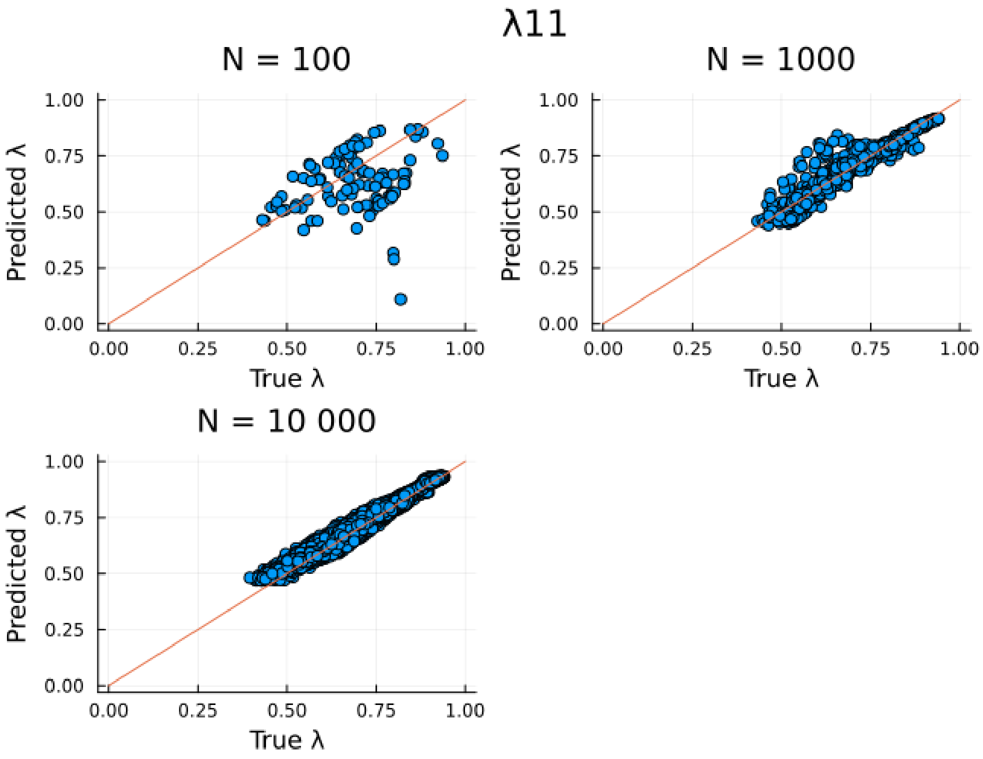

Fig. 2 True versus model predicted individual transition probabilities for different number of simulated individuals. Red line is the line of identity. N, number of individuals.

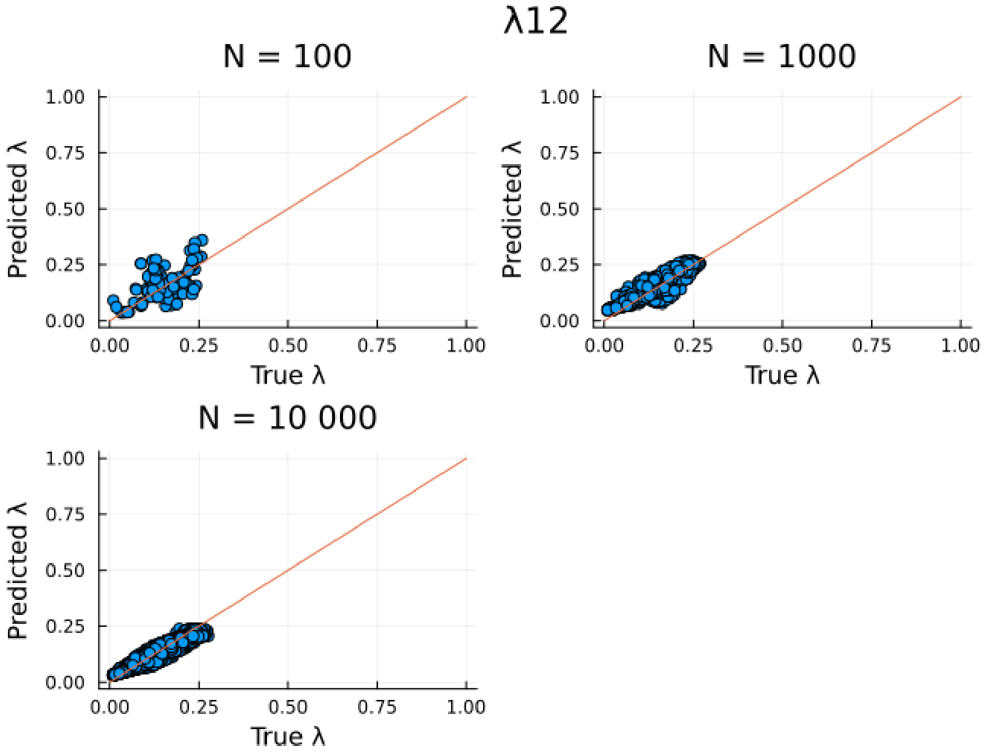

Fig. 3 True versus model predicted individual transition probabilities for different number of simulated individuals. Red line is the line of identity. N, number of individuals.

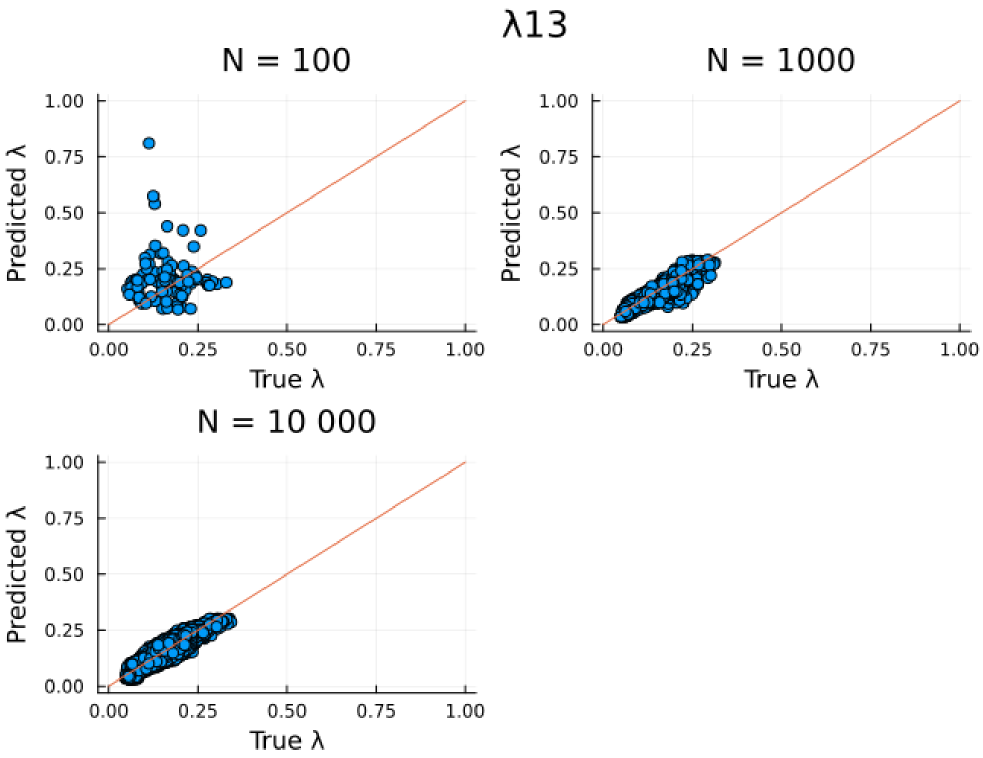

Fig. 4 True versus model predicted individual transition probabilities for different number of simulated individuals. Red line is the line of identity. N, number of individuals.

3 Performance depending on ANN size and depth

Table 1 Evaluation metrics for fitted ANN models with different number of nodes and hidden layers.

Model	Loss value	MSE λ_{11}	MSE λ_{12}	MSE λ_{13}
Two layers, 50 nodes	1265	0.153	0.058	0.095
Two layers, 100 nodes	1263	0.15	0.057	0.094
Three layers, 50 nodes	1265	0.151	0.057	0.095

MSE, mean squared error; λ_{mn}, probability of transition from state m to state n. Loss value is the minimized joint log-likelihood.
